# Nutrient and aflatoxin contents of traditional complementary foods consumed by children of 6–24 months

**DOI:** 10.1002/fsn3.621

**Published:** 2018-03-14

**Authors:** Emmanuel Oladeji Alamu, Therese Gondwe, Juliet Akello, Nancy Sakala, Grace Munthali, Mweshi Mukanga, Busie Maziya‐Dixon

**Affiliations:** ^1^ Food and Nutrition Sciences Laboratory International Institute of Tropical Agriculture (IITA) Ibadan Nigeria; ^2^ Plant Pathology Unit International Institute of Tropical Agriculture (IITA) Ibadan Nigeria; ^3^ Plant Protection and Quarantine Zambia Agriculture Research Institute (ZARI) Chilanga Zambia; ^4^ Food Science Research Center National Institute of Scientific and Industrial Research Lusaka Zambia; ^5^ Department of Agriculture Ministry of Agriculture Mulungushi House Lusaka Zambia

**Keywords:** aflatoxin, breastfeeding, complementary foods, porridge

## Abstract

The nutrient composition and safety of complementary foods have recently become areas of concern, especially with regard to aflatoxin contamination which has been found to adversely affect health outcomes. This study presents the nutrient and aflatoxin contents of complementary foods consumed by children (6–24 months) and infants and young child feeding practices of mothers from two districts in eastern and southern Zambia. A total of 400 mother–child pairs were recruited from Monze and Chipata districts, and data on breastfeeding and complementary feeding practices were collected twice at 3‐month interval using a structured questionnaire. Samples of two traditional complementary foods (Maize Nshima and Maize porridge) were collected from the mothers and analyzed for nutrient contents and aflatoxin contamination. The results showed that there is a high level of awareness on exclusive breastfeeding among mothers. Fat, protein, carbohydrate, and ash contents of Maize nshima from Chipata were significantly lower (*p* < .05) compared to those from Monze district except for starch and sugar. Monze mothers preferred to prepare a thicker Maize nshima and Maize porridge compared to their Chipata counterparts. The aflatoxin contamination showed that the Maize porridge samples from Chipata were the most contaminated with mean aflatoxin content of 5.8 ± 15.93 mg/100 g, while Maize nshima was the most contaminated of the two complementary foods from Monze districts with mean aflatoxin level of 3.8 ± 6.41 mg/100 g. There were significant (*p* < .05) positive correlations between fat and aflatoxin contents for Chipata samples (*r* = .12409) and for Monze samples (*r* = .13666). The traditional complementary foods studied were found to be low in fat and protein and high in aflatoxin contamination. Thus, it is imperative that best practices and interventions are designed and introduced to reduce the possible lethal health implications of consumption of such complementary foods by children under 5 years.

## 1 INTRODUCTION

Complementary foods are given to infants and children when the breastmilk is no longer enough to meet the nutritional requirements (WHO, 2013). The World Health Organization (WHO) recommends these foods to be started at once after a 6‐month period of exclusive breastfeeding. Appropriate and adequate complementary foods are essential for good physical and mental growth in infants and young children (Ijarotimi & Keshinro, 2013). Many products can be used as complementary foods, but the most widely consumed and nutritionally cost‐effective products in most of the developing countries are composite flours, which can be used to produce a gruel or porridge, typically made from a starchy staple (e.g., maize, rice, or wheat) and mixed in fixed ratios with a legume flour (e.g., roasted soya beans) and other ingredients. The introduction of proper complementary foods at proper time promotes good nutritional status and growth in infants and young children (Ijarotimi & Keshinro, 2013; Ogunlade et al., 2011). In Zambia, the introduction of complementary food usually starts between age 4 and 6 months, and maize is the common ingredient used to prepare semisolid maize‐based porridge called “maize porridge” or solid porridge called “Nshima.” Most often, these complementary maize‐based foods are blended with groundnuts (*Arachis hypogea* L.) to improve the nutritional value. The level of improvement, as well as the proportion of water to flour, is not standardized, so that children tend to receive gruels that are either too diluted or too concentrated to meet the daily recommended dietary allowances. In addition, because of inappropriate processing technology, the gruels have significant levels of contaminants and antinutritional factors, which further limit nutrient absorption by the young child (Oumarou, Ejoh, Ndjouenkeu, & Tanya, 2005). Many research studies have reported the nutrient composition of complementary foods (including the fortified blended complementary foods) in different part of the world, most especially in developing countries. Adepoju and Etukumoh (2014) evaluated the nutrient composition of four used complementary foods (CFs) in two local government areas (LGAs) of Akwa Ibom State, Nigeria. About 100 g portion of the CFs contained between 2.52 and 6.70 g crude protein, 1.26 and 7.23 g crude lipid, and 8.16 and 13.97 g carbohydrates and yielded up to 415.57 kcal of energy. Ijarotimi and Keshinro (2013) also evaluated nutritional quality of complementary foods using popcorn, Bambara groundnut, and African locust beans and reported protein contents ranging from 26.87 to 20.87 g/100 g for various blended complementary foods. Moreover, Anigo et al. (2010) tried to formulate low‐cost, nutritive complementary foods using malted maize, millet, and sorghum with groundnut and soybean. Nutrient qualities of gruel of the formulations thus prepared were evaluated. The ash content ranged from 1.03 to 2.54 g/100 g with the crude protein ranging from 6.37 to 7.88 g/100 g. Gibson et al. (1998) reported that complementary foods should give 25%–50% of total daily requirements for protein and that most of the twenty‐three plant‐based complementary foods consumed in developing countries appear to meet the estimated daily nutrient needs (per day; per 100 kcal) from complementary foods for protein. Of recent, asides from nutrient quality, another area of concern is food safety with regard to aflatoxin contamination which has been found as a threat to improved health and economic status in sub‐Saharan Africa (Abt Associates, 2012; Gong et al., 2004; Kaaya & Warren, 2005; Pritchard, Ortiz, & Shekar, 2016) especially in Zambia (Akello et al., 2015). Mycotoxins are natural contaminants formed as secondary metabolites by toxigenic fungi in the field and/or during storage under suitable physical, chemical, and biological factors (Shepard, 2008). Of the 400 mycotoxins produced, agriculturally important fungal toxins are deoxynivalenol, zearalenone, ochratoxins, fumonisins, and aflatoxins (Miller, 1995; Wagacha & Muthomi, 2008). Aflatoxins are produced by *Aspergillus flavus and Aspergillus parasiticus* in both field and storage. Infection is most common after the kernels have been damaged by insects, birds, mites, hail, early frost, heat and drought stress, windstorms, and other unfavorable weather (Jacobsen, 1993). Unfortunately, the presence of aflatoxins in food is often overlooked in Africa due to varying factors varying from public ignorance about their existence to lack of regulatory mechanisms. Exposure to aflatoxins in humans is usually through direct ingestion of contaminated foods of plant origin (such as maize and groundnuts) and through ingestion from meat and milk/milk products (WHO, 1979). At high exposure levels, aflatoxins can cause acute toxicity and potentially death in humans. The liver is the principal organ affected, but high levels of aflatoxin have also been found in the lungs, kidneys, brains, and hearts of individuals dying of acute aflatoxicosis. Acute necrosis and cirrhosis of the liver is typical, along with hemorrhaging and edema. Aflatoxins are quite stable compounds and survive relatively high temperatures with little degradation. Other factors influence their heat stability, such as moisture level and pH, but heating or cooking processes cannot be relied upon to destroy aflatoxins. Most complementary foods are cereal‐dependent and are thus susceptible to aflatoxin contamination which has been confirmed by evidence in sub‐Saharan countries such as Benin (Gong et al., 2004), Gambia (Turner et al., 2007), Uganda (Asiki et al., 2014), and Tanzania (Routledge, Kimanya, Shirima, Wild, & Gong, 2014; Shirima et al., 2013). Most of the studies in these populations set up some disease outcomes to this contamination. In Zambia, maize contributes as much as 49.4% of the dietary calories intake (FAO, 2013). The reliance on maize‐ and groundnut‐based meals during a child's weaning stage could increase exposure to food‐borne mycotoxins particularly aflatoxins. Thus, these food mixes may be resulting in aflatoxin exposure beyond the provisional greatest tolerable limit (2 ppb/day). High consumption of maize‐ and groundnut‐based complementary meals among infants exposes them to aflatoxins. There is evidence to show that aflatoxin may increase micronutrient deficiencies in children (Obuseh et al., 2011). Little is known on aflatoxin contamination of complementary foods fed to children between 9 and 24 months in Zambia. Kachapulula, Akello, Bandyopadhyay, and Cotty (2017) re‐emphasized this threat for children by giving evidence of aflatoxin contamination in both maize and groundnuts. Finished food products should have less than 0.1 ppb of aflatoxin, EU and USA standards for corn/soy/groundnut blends for young children (MIYCN Working Group, 2009). There are various causes of inadequate and unsafe complementary foods that contribute toward malnutrition in Zambia. Among them include the following: use of unsafe drinking water, poor infant, and child feeding practices which are often associated with lack of knowledge for the mothers and caregivers on how to prepare nutritious foods; the content of the diet is mostly dominated by carbohydrates with less fruit, vegetables, legumes, and animal protein; limited access to enough quality foods, limited knowledge on how to prepare nutritious foods. The influence of high levels of dietary contamination in complementary foods used for weaning Zambian children is not clear. Hence, child aflatoxin exposure in Zambia could be an under‐evaluated risk factor. It is usually an advantage to analyze samples as they were from the households and as being fed to the children, to have results closer to reality when compared with laboratory prepared samples. Hence, this study focused on collecting samples of complementary foods given to the children in the households sampled. As quality complementary foods are a necessity to supplement breastfeeding of infants, it is thus important to probe popular local recipes to find nutrient and aflatoxin contamination levels. This study aimed at evaluating the nutrient and aflatoxin contents of complementary foods consumed by children (6 to 24 months) and infants and young child feeding practices of mothers from two districts in eastern and southern Zambia.

## 2 METHODOLOGY

### 2.1 Study design

The study combined both a cross‐sectional design to interview the respondents on breastfeeding and complementary feeding practices and used a laboratory‐based analytical design for the analysis of proximate parameters (protein, ash, fat, crude fiber, and carbohydrate) and aflatoxin contamination in sampled complementary foods.

### 2.2 Study location and sampling of respondents

A total of 400 mother–child pairs from selected eight agricultural camps in each of the two districts (Monze and Chipata districts) were randomly selected for this study. A minimum total of 25 mother–child pairs were selected from each camp based on the average numbers of mother–child pairs registered at the camp clinics. Two hundred (200) mothers with children 6 to 24 months from the selected households in each of the two districts were recruited into the study. An agricultural camp is a small unit in the agricultural sector where farmers are grouped around agricultural extension service provision with an extension officer.

### 2.3 Selection and training of research assistants

A total of 20 research assistants (12 females and 8 males) from the two districts were selected and trained for the survey and collection of complementary samples for 8 days. The seasoned facilitators were from Nigeria and Zambia. The training were into morning and afternoon sessions, which involved both lectures and practicals.

### 2.4 Pretesting and revision of survey questionnaires

The pilot study was conducted after the training to pretest the instrument and methodology designed for the study. A neutral camp, “Siakasenke,” was used for the pretesting, and 25 households were sensitized and participated. The questionnaires were administered, and complementary samples were collected by the trained personnel. The instruments were revised after analyzing the pilot survey data.

### 2.5 Assessment of Infant and child feeding practices

A survey of participant mothers using structured questionnaire was conducted to collect the breastfeeding and complementary feeding practices information for two times at 3‐month interval.

### 2.6 Sampling of complementary food

The trained research assistants collected each complementary sample w into a well‐labeled plastic container with snap lid (500 mL size) and all transferred into freezer (‐4 degrees centigrade) until being analyzed for nutrients and aflatoxin contents. The samples were collected twice at the interval of 3 months.

### 2.7 Chemical properties determinations

Moisture, ash, fat, and crude protein contents were determined using the methods described by Alamu, Maziya‐Dixon, Popoola, Gondwe, and Chikoye (2016) and the Association of Official and Analytical Chemist (AOAC) (2005). The loss in weight after drying for 24 hr in Fisher Scientific Isotemp^®^ Oven model 655F was recorded as moisture content. The ash content was estimated after moisture, and all organic constituents were burned off at 600°C. Crude fat was extracted from the sample with hexane, and the solvent evaporated off to get the fat. Protein content was determined by the Kjeldahl method using Kjeltec^™^ Model 2,300, as described in Foss Analytical Manual AB. (2003). A conversion factor of 6.25 was used to convert from total nitrogen to percentage crude protein. Total carbohydrate (CHO) was calculated by difference using the method described by Merrill and Watt (1973) as follows: %CHO = 100−(%Protein+%fat + %Ash + % Crude fiber +% Moisture).

### 2.8 Aflatoxin determination

The REVEAL Q+ FOR AFLATOXIN GREEN test method provided by the Neogen Corporation was used to determine the aflatoxin level (Neogen, 2015). The test provides quantitative analysis for the presence of aflatoxin, using nonhazardous, water‐based extraction solution along with an aflatoxin‐antibody particle complex coated test strip and the Neogen AccuScan Gold reader. About 50 g sample was blended in 100 mL of 65% ethanol for 1 min in a high‐speed blender (Waring Commercial, Springfield, MO, USA), and the content was transferred to a conical flask. The blender was rinsed with 150 mL of 65% ethanol, and the content was added to the flask. The content was mixed by swirling, shaken on rotary shaker at maximum speed for 3 min, and the content was filtered through a Whatman No. 1 filter paper. The sample extract (100 μL of sample) was mixed with the Reveal Q^+^ sample diluent (500 μL of diluent). A Reveal Q^+^ test strip was dipped in 100 μL of the mixture for 6 min, removed, and inserted in the reader. The device has a quantification range of 2 to 150 ng/g with a detection limit of 2 ng/g. Samples containing over 150 ng/g were diluted with 65% ethanol, and new readings were taken. To validate the readings, aflatoxin standard reference materials were used. Mycotoxin reference materials with known toxin level were sourced from Neogen and used to validate the results after every run of 25 samples. The calibration of equipment was performed each time a new test kit was opened, and the Neogen machine was calibrated as per protocol from the manufacturer.

### 2.9 Ethical clearance

An ethical clearance approval was obtained from The University of Zambia Biomedical Research Ethics Committee (UNZABREC) with Assurance No. FWA00000338 and approval number IRB00001131 of IORG0000774, and a final authority was obtained from the Ministry of Health. The mother of the selected child was informed about the nature of the study. Subject participation in the study was voluntary with informed consent requested from head of the household.

### 2.10 Statistical analysis

All laboratory analyses were carried out in duplicate, and the mean ± standard deviation of the results from the experiment was calculated and analyzed using Statistical Analysis System (SAS) software package version 9.3. The Duncan's multiple range test was used to separate the differences in the mean scores at significant level of *p* < .05.

## 3 RESULTS AND DISCUSSION

Infant child feeding practices of mothers from Monze and Chipata districts, nutrient content of traditional complementary foods of their infants, and aflatoxin contamination of these traditional foods are presented in Tables 1, 2, 3, 4, 5, 6.

**Table 1:** Distribution of awareness levels on exclusive breastfeeding and practices of complementary feeding

	Categories	Chipata	Monze
		Percent (*N*)	Percent (*N*)
Awareness	Yes	52.55 (381)	39.86 (289)
	No	4.55 (33)	3.03 (22)
Introduction to solids	0 months	2.9 (21)	1.93 (14)
	3 months	1.93 (14)	1.52 (11)
	6 months	39.03 (283)	33.52 (243)
	Others	13.1 (95)	5.79 (42)
Period last child was breastfed	0 months	0.62 (4)	0.93 (6)
	1 months	0.47 (3)	0 (0)
	2 months	0.62 (4)	0.16 (1)
	3 months	2.33 (15)	1.71 (11)
	4 months	3.88 (25)	1.4 (9)
	5 months	4.81 (31)	2.48 (16)
	6 months	42.48 (274)	38.14 (246)

**Table 2:** Comparison of proximate composition of complementary food (Nshima) in Chipata and Monze

Parameter (g/100 g)	Chipata (*N* = 161)				Monze (*N* = 140)			
	Mean ± *SD*	Min	Max	CV	Mean ± *SD*	Min	Max	CV
MC	26.6 ± 3.58a	15.2	34.8	13.4	32.4 ± 2.07b	26.5	35.4	6.4
DM	73.1 ± 3.08a	63.4	83.2	4.2	67.6 ± 2.07b	64.6	73.5	3.1
ASH	0.2 ± 0.23a	0	2.2	94.5	0.7 ± 0.36b	0.3	1.6	50.7
FAT	0.1 ± 0.11a	0	0.9	75.4	0.3 ± 0.14b	0	0.6	40.7
PROTEIN	2.5 ± 0.84a	0.1	8.7	33.4	2.6 ± 0.41a	1.8	3.4	16
SUGAR	1.1 ± 2.26a	0	23.3	210.9	0.8 ± 0.65a	0.1	2.2	81.7
STARCH	23.2 ± 5.47a	13.6	38.5	23.6	28.8 ± 5.7b	18.3	40	19.8
CHO	24 ± 3.15a	14.4a	34	13.1	28.8 ± 2.22b	22.7	31.9	7.7

CHO, total carbohydrate; DM, dry matter; MC, moisture content.

Rows with same letters are not significantly different at *p* = 0.05.

**Table 3:** Comparison of proximate composition of complementary food (Maize‐based porridge) in Chipata and Monze

Parameter (g/100 g)	Chipata (*N* = 60)				Monze (*N* = 41)			
	Mean ± *SD*	Min	Max	CV	Mean ± *SD*	Min	Max	CV
MC	78.8 ± 4.75a	70.4	88.6	6	82.5 ± 1.53a	80.9	84.2	1.9
DM	21.2 ± 4.75a	11.4	29.6	22.4	17.5 ± 1.53a	15.8	19.1	8.7
ASH	0.4 ± 0.32a	0.1	1.1	83.7	0.2 ± 0.07b	0.1	0.3	32.7
FAT	0.2 ± 0.09a	0	0.3	58.7	0.3 ± 0.19a	0.2	0.5	56.9
PROTEIN	2 ± 0.82a	1	3.7	41.8	2.2 ± 0.06a	2.1	2.2	2.7
SUGAR	1 ± 0.62a	0.1	2.5	62.4	0.1 ± 0.08b	0	0.2	78.9
STARCH	21.5 ± 4.02a	14.7	29.4	18.7	16 ± 0.29b	15.8	16.4	1.8
CHO	18.7 ± 4.71a	10	26.1	25.3	14.8 ± 1.32b	13.3	16.2	8.9

CHO, total carbohydrate; DM, dry matter; MC, moisture content.

Rows with same letters are not significantly different at *p* > 0.05.

**Table 4:** Mothers’ perspective on quality characteristics that determine type of complementary foods to feed the children

Profile	Response	Chipata	Monze
		Percent (*N*)	Percent (*N*)
Taste	Yes	5.24 (38)	33.66 (244)
	No	52 (377)	9.1 (66)
Cost	Yes	4.97 (36)	11.31 (82)
	No	52.28 (379)	31.45 (228)
Availability	Yes	27.17 (197)	28 (203)
	No	30.07 (218)	14.76 (107)
Age	Yes	20.41 (148)	15.86 (115)
	No	36.83 (267)	26.9 (195)
Preparation	Yes	2.21 (16)	4.97 (36)
	No	55.03 (399)	37.79 (274)
Others	Yes	0.28 (2)	1.66 (12)
	No	56.97 (413)	41.1 (298)

**Table 5:** Aflatoxin level (mg/100 g) of complementary foods from Chipata and Monze

Location	Food type	*N*	Mean	Median	*SD*	Min	Max	CV
Chipata	Nshima	161	2.2cd	1.4	3.96	0.4	30	55.56
	Porridge	60	5.8a	1.9	15.93	0.1	104	36.41
Monze	Nshima	140	3.8b	1.5	6.41	0.3	33.6	59.28
	Porridge	14	2.0d	1.9	1.18	0.5	3.6	169.49

Columns with different letters are significantly different at *p* < .05.

**Table 6:** Correlation between chemical properties and aflatoxin content of complementary foods sampled from Chipata and Monze districts

	MC	Ash	Fat	Protein	Sugar	Starch	CHO	Energy
Chipata district								
Aflatoxin content	0.11137	−0.01679	0.12409^a^	−0.01877^a^	0.08042	−0.10615	−0.09856	−0.11379
Monze district								
Aflatoxin content	−0.19283	−0.05785	0.13666^a^	−0.40105^a^	0.23213	0.04349	−0.10470	−0.18787

CHO, total carbohydrate; MC, moisture content.

a Significance at *p* < .05.

### 3.1 Awareness and practices of exclusive breastfeeding by mothers

The mothers were found to have a high level of awareness on exclusive breastfeeding made possibly due to increased contact with the community healthcare providers and attending the scheduled postantenatal sessions. Less than 10 percent of all respondent were unaware of the definition and advantages of exclusively breastfeeding infants for at least 6 months (Table 1). This result holds an advantage for care of the infant, because a mother who has high awareness of exclusively breastfeeding is more likely to start and practice proper exclusive breastfeeding (Jiang et al., 2012). The period of introduction of “solid food” by mothers also followed a similar pattern to that of awareness of exclusively breastfeeding practices. About 73% of the mothers both from Chipata (39.03%) and Monze (33.52%) introduced solid foods just after 6 months. Exclusive breastfeeding is recommended up to 6 months of age by WHO, with continued breastfeeding along with appropriate complementary foods up to two years of age or beyond. More than 76% of the respondents in Chipata district breastfed their last child for at least 6 months. A similar high number (246 mothers) of respondents from Monze also continued breastfeeding for at least 6 months (Table 1). This study showed that most mothers from both districts complied with WHO recommended period of exclusive breastfeeding. It is clear that timing of the introduction of complementary foods was proper; however, the quality of the foods may be found to be the cause of short‐term and long‐term outcomes of undernutrition and overnutrition (Hussein, 2005; Muniandy, Allotey, Soyiri, & Reidpath, 2016) being recorded in these areas. There was higher number of Chipata mothers with greater awareness and longer practice of exclusively breastfeeding compared to their Monze counterparts. This could be due to the difference in knowledge in nutrition education, attitude, and culture among mothers, and these factors have been found to be strongly associated with infant and child care practices (Katepa‐Bwalya et al., 2015). This difference presents a need for public health nutrition intervention in Monze to sensitize the mothers to improve their infant care practices. An increase in educational level of the mother or caregiver has been found to improve practice of proper and adequate infant care practices (Katepa‐Bwalya et al., 2015; Runsewe‐Abiodun, Bondi, Alabi, & Taqi, 2016).

### 3.2 Nutrient contents of maize‐based complementary food samples

The basic nutrient contents of Nshima and Maize porridges—both the commonest complementary foods—sampled from Chipata and Monze are presented in Tables 2 and 3. The results showed that the fat, protein, carbohydrate, and ash contents of Nshima from Chipata were significantly lower (*p* < .05) compared to those from Monze except for starch and sugar that were nonsignificantly (*p* < .05) lower. This shows that the nutrient composition of Nshima as complementary food from the two districts was different, and this could be due to nonstandardization of preparation and the types of maize used as raw material. This agrees with what Oumarou et al. (2005) reported, the level of proportion of water to flour and processing technology affected the quality of complementary foods. The values of protein, fat, and ash reported in this study were in close agreement with what Adepoju and Etukumoh (2014) reported for the four used complementary foods (CFs) in two local government areas (LGAs) of Akwa Ibom State, Nigeria. The mean moisture content of Nshima from Chipata was 26.6 ± 3.58 g/100 g and lower than those sampled from Monze (32.4 ± 2.07 g/100 g). This shows that the nshima being fed to children from Chipata was a little bit thicker than that of Monze, while the starch and carbohydrate contents of porridge from Chipata were significantly (*p* < .05) higher compared to those from Monze. The results presented in Tables 2 and 3 show an obvious difference in the preparation methods of the same food across the two districts. This is clear in the moisture contents of the two foods which show that Monze mothers preferred to prepare a thicker Nshima and Maize porridge compared to their Chipata counterparts. This is concordant with the findings of Shindano (2007). However, the resulting higher dry matter in the thicker foods did not necessarily translate into higher nutrient density in the sampled foods.

In general, the nutrient content of the traditional complementary samples from Zambia was very low compared to the blended complementary samples reported in the literature and the recommended WHO nutrient level for complementary foods. According to Ijarotimi and Keshinro (2013), the protein content should range from 26.87 to 20.87 g/100 g for various blended complementary foods. Anigo et al. (2010) suggested the ash content ranged from 1.03 to 2.54 g/100 g with the crude protein ranging from 6.37 to 7.88 g/100 g for blended complementary food samples. Thus, the complementary foods from Zambia need improvement in their nutrient contents to meet the nutrient daily requirements of the weaning children. However, Gibson et al. (1998) reported that fortified plant‐based complementary foods gave 25%–50% of total daily requirements for protein.

### 3.3 Mothers perspective on quality characteristics of maize‐based complementary foods

Responses to questions on quality characteristics that decide the type of complementary foods to feed the children are grouped into taste of the food item, cost of preparing the food, availability of ingredients for making the food, the age of the child, and the method of preparation (Table 4). The results showed that most mothers from both districts did not consider the cost (84% of mothers) and method of preparation (93% of mothers) of complementary foods to be major characteristics. This suggests that these characteristics do not affect the choice of food prepared by the mothers for their growing infants. This is similar to the observation made by Chisenga, Siame, Baisley, Kasonka, and Filteau (2011) who found that lower income women scored higher in giving adequate infant feeding that those with higher economic status. This noneffect of economic status suggests that intention to give care supersedes availability of resources. However, there was no clear difference for the results for age as a factor, and mothers from Chipata who responded with negative response (36.83%) compared to positive response (20.41%) were like the response of negative (26.9%) and positive (15.86%) for Monze mothers (Table 4). This implies that irrespective of the age, the choice of complementary foods given to the child did not vary. Taste of the foods, however, had influence on the preference of the complementary food chosen by the mothers. While mothers from Monze were more influenced by the taste of the food, their counterparts from Chipata were more flexible about the taste of the food. A similar trend was observed with the factor “availability of materials” for preparation of the complementary foods. This observed difference of opinion could be linked to diverse cultural contexts which are distinct across the districts as reported by Alaofe et al. (2012) in a study of households of northern provinces of Zambia.

### 3.4 Aflatoxin contents of maize‐based complementary food samples

Table 5 shows the aflatoxin content of commonest complementary foods (Nshima and Maize porridge) among mothers in Zambia. Porridge (5.8 ± 15.93 mg/100 g) from Chipata was the most contaminated of all samples, while Nshima (3.8 ± 6.41 mg/100 g) was the most contaminated of the two complementary foods from Monze. The mean aflatoxin concentrations of studied complementary foods were much higher than the acceptable limits for crops in Zambian populations which are 2 ppb. The mean values are also higher than allowed World Food Programme standards for finished foods (MIYCN Working Group – Subgroup on Formulation Guidelines, 2009). Notable is the observation of some food samples which were as high as 104 mg/100 g (Porridge) in Chipata and 33.6 mg/100 g (Nshima) in Monze. With consumption of aflatoxins contaminated food being associated with pathology of several liver diseases (Williams et al., 2004; Reddy & Raghavender, 2007) and has been highlighted as a cause of stunting in under‐5 children (Gong et al., 2004; Turner et al., 2007). It is necessary to design and introduce most economical and practical interventions to reduce the possible lethal health implications of high exposure of aflatoxin for mothers and children in Chipata and Monze districts.

### 3.5 Correlation analysis of parameters of maize‐based complementary food samples

Correlation coefficients of the relationships between the chemical properties and aflatoxin levels of the studied sampled foods are presented in Table 6. The moisture content, fat, and sugar of complementary samples from Chipata showed a nonsignificant (*p* > .05) positive correlation with aflatoxin levels but ash, protein starch, and total carbohydrate showed a weak negative correlation. This suggests that food samples with high aflatoxin contamination showed poor nutrient level, and the consumption of highly contaminated samples would mean poor consumption of essential nutrients and has double negative effects on the children nutritional status of the children. However, only sugar and starch showed weak and nonsignificant (*p* > .05) positive correlation for Monze complementary samples. There was significant (*p* < .05) positive correlation between fat and aflatoxin for Chipata samples (*r* = .12409) and for Monze samples (*r* = .13666). However, there was significant (*p* < .05) negative correlation for protein (Chipata samples, *r* = −.01877, Monze, *r* = −.40105). These results agree with the findings of Zubair et al. (2011), who reported that the interaction of aflatoxin with proximate composition and fatty acids showed that B1 was positively correlated (*r* > .05) with protein, ash, and fiber while negatively correlated (*r* < −.05) with fat and oleic acid. It could be concluded that the toxin production efficiency of *Aspergillus flavus* was dependent on biochemical composition.

## 4 CONCLUSION

In conclusion, the differences in the food preparation and feeding practices of mothers from these two districts could help design interventions and develop nutrition programmes aimed at the targeted populations. The high levels of aflatoxin found in the complementary foods call for newer emphasis to be placed on the way complementary foods are prepared and on the need to be given in a safe manner, meaning that measures must be taken to minimize the risk of contamination with pathogens. The traditional complementary foods studied were seen to be low in fat and protein which are needed for growth and development; henceforth, there is an urgent need for nutrition education on the blending of these complementary foods with legumes to improve the nutritional value of these maize‐based foods. In addition, care should be taken on the choice of food legumes as a protein intake, as most complementary foods are based on plant sources, whose bioavailability is inferior to protein from animal sources.

## CONFLICT OF INTEREST

The authors declare that they do not have any conflict of interest.

## ETHICAL STATEMENTS

Ethical Review: This study was ethically reviewed and approved by University of Zambia Biomedical Research Ethics Committee (UNZABREC). Technical support was given by the Zambian Ministry of Health.

Informed Consent: Written informed consent was obtained from the mothers who took part in this study.
